# A multicenter randomized controlled trial to assess the efficacy of cancer green therapy in treatment of stage IIIb/IV non-small cell lung cancer

**DOI:** 10.1097/MD.0000000000021626

**Published:** 2020-08-14

**Authors:** Manqiang Sun, Tian Zhou, Xueni Fang, Dan Wang, Haoyue Pang, Yu Chen, Kaiwen Hu

**Affiliations:** Dongfang hospital Beijing University of Chinese Medicine, No.6 Community 1 Fangxingyuan, Fengtai District, Beijing, China.

**Keywords:** chemotherapy, cryoablation, randomized controlled trial, stage IIIB/IV non-small cell lung cancer, traditional Chinese medicine formula

## Abstract

**Background:**

Chemotherapy is the main therapy for stage IIIB/IV non-small cell lung cancer (NSCLC). However, the 5-year survival rate is 6%. Cancer Green Therapy is a novel therapy in China, which refers to cryoablation combined with traditional Chinese medicine (TCM) formula. Our previous retrospective analysis showed that patients with NSCLC had longer survival time and better quality of life after receiving cryoablation combined with TCM formula, compared with patients who received chemotherapy alone.

**Methods:**

This study is a multicenter, randomized, controlled clinical study. The experiment will be carried out in 6 hospitals at the same time, and a total of 450 cases of participants will be randomly assigned to the experimental group and the control group (n = 225). The experimental group will be given cryoablation and 28-days TCM formula, and the control group will be given 4 cycles chemotherapy. After 30 months of follow-up, the efficacy and safety of cryoablation combines with TCM formula in patients with stage IIIB/IV NSCLC will be observed. The primary outcome is overall survival. The secondary outcomes include progression-free survival, objective response rate, and quality of life. We will also conduct a safety evaluation of the treatment at the end of the trial.

**Discussion:**

This multicenter, randomized, controlled clinical study not only provides data on the efficacy and safety of cryoablation combined with TCM formula, but also provides a novel treatment strategy for clinicians and advanced NSCLC patients.

## 1 Introduction

Lung cancer is the most common type of cancer and the leading cause of cancer death.^[1]^ Non-small cell lung cancer (NSCLC) accounts for 75% to80% of lung cancer, and the 5-year survival rate is about 15%.^[2]^ The main pathological types of NSCLC are adenocarcinoma (32% – 40%), squamous cell carcinoma (25% –30%), and large cell carcinoma (8% –16%).^[3]^ About 70% of lung cancer patients are at an advanced stage when they are first diagnosed, and surgery therapy isn’t available for which. Chemotherapy, radiotherapy, targeted therapy, and immunotherapy can be selected as treatments for stage IIIB/IV NSCLC, platinum-based doublets chemotherapy is the main option among which. However, the effective rate of each chemotherapy regimen varies from 20% to 30%, and progress-free survival (PFS) is only a few months.^[4–6]^ Also, adverse events such as marrow suppression, nausea, vomiting can decrease the quality of life (QOL) of patients.^[7]^ It is necessary to find an effective and safe treatment for advanced NSCLC.

Cryoablation is a novel surgical therapy for benign and malignant tumors, especially for unresectable tumors. It's a minimally invasive treatment in which the tumor is frozen and destroyed by cryoprobes, and the process lasts about 20 minutes. Therefore, cryoablation has the advantages of highly targeted, more repeatability, less trauma, and fewer side effects.^[8,9]^ It is widely used in liver cancer, kidney cancer, prostate cancer, and lung cancer.^[10–14]^ In a study, 50 patients with advanced lung cancer were treated with argon-helium knife ablation. The treatment effect was relatively ideal, with a median survival time of 19 months, a 1-year survival rate of 87.2%, and a 2-year survival rate of 22.4%.^[15]^ Some studies have suggested that argon-helium knife cryoablation is a better minimally invasive treatment for elderly lung cancer patients, which has a slight impact on the respiratory function of patients, prolong the survival time of patients, and improve the related symptoms caused by tumors.^[16]^

Traditional Chinese medicine (TCM) has certain advantages in the treatment of advanced NSCLC. When combined with chemotherapy and radiotherapy, it can enhance long-term efficiency and decrease side effects of them, such as bone marrow suppression, nausea, vomiting, and radioactive inflammation. In addition, TCM can promote postoperative recovery and prevent tumors’ relapse and metastasis.^[17]^ As for those who can’t receive surgical therapy, chemotherapy, and radiotherapy, TCM can improve clinical symptoms and the QOL of patients. What's more, many studies report that TCM can inhibit or stabilize the development of tumors, help patients achieve “survival with tumor” .^[18]^ However, many studies of TCM have problems with sample size and follow-up visits. Thus, they need to be further improved.

Initially, many elderly patients with advanced lung cancer chose Cancer Green Therapy (cryoablation combined with TCM) for better QOL. However, we found that the survival was longer than that we had expected during the follow-up visit. Later, we conducted a retrospective analysis and screened 186 patients who underwent cryoablation plus TCM in our department between 2005 and 2013. 119 cases were followed up in 2014. Among them, 82 cases were advanced NSCLC patients, the average overall survival (OS) of them is 18 months, almost double as that in NCCN guidelines (8–10 months).^[19,20]^

The present multi-center, prospective, randomized controlled trial is designed to investigate the efficacy and safety of Cancer Green Therapy (cryoablation combined with TCM), setting chemotherapy as the control treatment. We hope that, through this rigorously designed study, we can provide scientific and objective assessments for the efficacy and safety of cryoablation combined with TCM for stage IIIB/IV NSCLC patients and possibly offer the world an alternative treatment.

## 2 Methods and design

### 2.1 Study design

**Figure 1:**
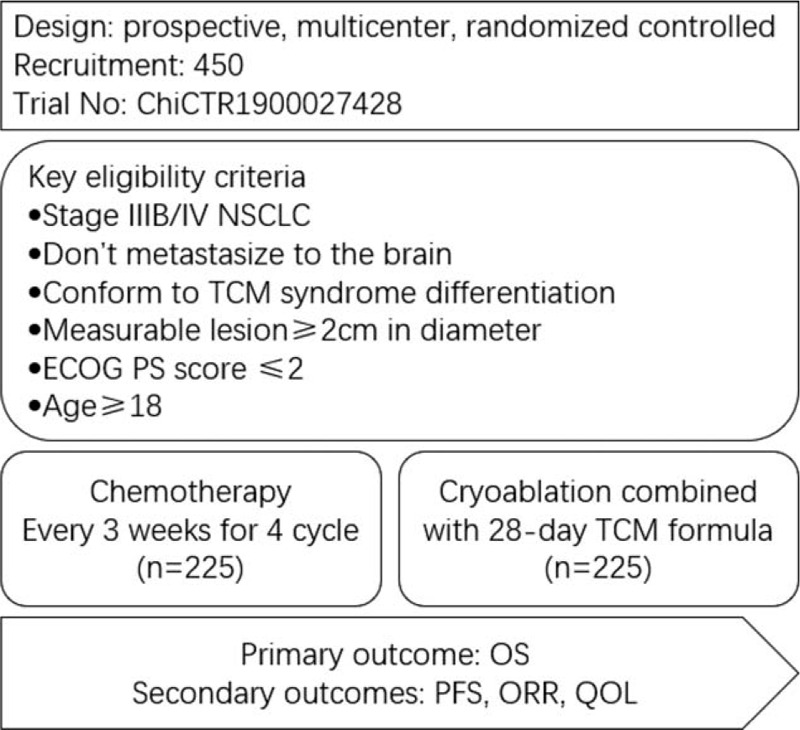
Overview of the Study Design. AEs = adverse events, ECOG = Eastern Cooperative Oncology Group, NSCLC = non-small cell lung cancer, ORR = objective remission rate, OS = overall survival, PFS = profession-free survival, PS = performance status, QOL = quality of life, TCM = traditional Chinese medicine.

The trial is a multi-center, prospective, randomized controlled trial. The flowchart of the study is presented in Figure 1.

### 2.2 Ethical issues

The clinical trial has been registered on the Chinese Clinical Trial Registry (ChiCTR1900027428). The study protocol (version 2.0 in December 2018) has been approved by the research ethics committee of Dongfang Hospital Beijing University of Chinese Medicine (JDF-IRB-2019030103). The research ethics committee will also be responsible for supervising all procedures of the study, including participant recruitment, randomization, conduction, and data storage. In case of any changes to the study protocol, we will submit a written application to the research ethics committee. They will decide whether it is acceptable to make the change.

### 2.3 Recruitment

A total of 450 eligible participants will be recruited from 6 hospitals in different parts of China: Dongfang Hospital, Beijing Hospital of Chinese medicine, China-Japan Friendship Hospital, Beijing Hospital, Longhua Hospital, and Hubei Hospital of Chinese Medicine. Both inpatients and outpatients will be screened for participation from March 2019.

### 2.4 Informed consent

Potentially eligible participants will be invited to take part in our study. Before enrollment, researchers will thoroughly introduce the detailed procedures of the study to the participants. All questions and concerns raised about the study will be addressed at length. Participants will also be informed of the probable benefits and potential risks and assured that participation is entirely voluntary. Participants who meet all the inclusion criteria and none of the exclusion criteria will be enrolled after providing written informed consent. The personal information of all the participants will always be kept confidential.

### 2.5 Participants

#### 2.5.1 Inclusion criteria

Participants meeting all the following criteria will be enrolled:

(1) Histologically or cytologically confirmed NSCLC and clinical-stage IIIB or IV; (2) Conform to TCM syndrome differentiation;
(2) Lesion ≥ 2 cm in diameter;
(3) Eastern Cooperative Oncology Group score ≤ 2;
(4) Expected survival ≥ 3 months;
(5) Age ≥ 18;
(6) Participate the trial voluntarily and sign written informed consent.

#### 2.5.2 Exclusion criteria

Participants meeting any 1 of the exclusion criteria will not be enrolled:

(1) Radiation therapy, chemotherapy, targeted therapy or immunotherapy within a month;
(2) Brain metastases;
(3) Inflammation around the lesion, or skin infection and ulceration at the puncture site;
(4) Severe pulmonary fibrosis;
(5) Severe bleeding tendency, platelet count < 50 × 10^9^/L and severe coagulation disorders;
(6) Malignant pleural effusion;
(7) Pregnant and lactating women; severe dysfunction of liver, kidney, heart, lung; severe anemia, electrolyte disturbance, and malnutrition; severe systemic infection and high fever (T > 38.5°C);
(8) HIV antibody positive; immunodeficiency diseases;
(9) History of organ transplantation;
(10) Enrolled in other clinical trials within 3 months.

### 2.6 Randomization and allocation concealment

The participants will be randomized at 1:1 (225 in each group) by researchers in Peking University clinical research institute (PUCRI). The Red Cap system of PUCRI will generate random allocation sequences based on the basic information (name, age, gender) of participants and feed back to researchers in the participating centers through the network.

### 2.7 Blind

In this study, researchers and patients will not be blinded due to the different treatment regimens. However, evaluators and statistical analysts will be blind to patient enrollment and treatment.

### 2.8 Interventions

Participants allocated to the study group will receive cryoablation and TCM syndrome differentiation and treatment, while chemotherapy will be applied in the control group.

Cryoablation therapy will refer to Expert Consensus for Thermal Ablation for Primary and Metastatic Lung Tumors (2017 Edition).^[21]^ Cryoablation will start in 2 weeks of randomization. TCM syndrome differentiation and treatment will be based on guidelines in the TCM Diagnosis and Treatment Scheme for Lung Cancer (2017) (Table 1). TCM formula will be made into water-soluble granules. Patients will dissolve them with warm water for a drink in the morning and evening for 28 days after cryoablation.

**Table 1:**
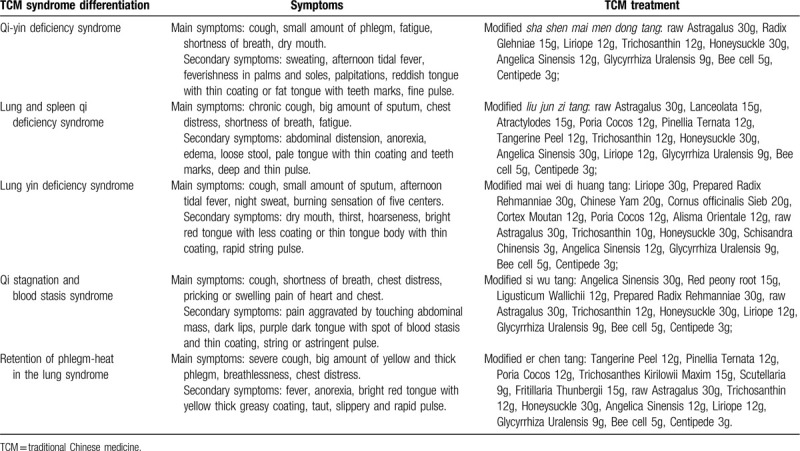
TCM Syndrome Differentiation and Treatment.

Participants in control group will receive 1 of following 5 platinum-based chemotherapy regimens. Each chemotherapy cycle has 3 weeks, lasting 4 to 6 cycles.

(1) NP regimen: vinorelbine 25 mg/m^2^ on days 1 and 8 and cisplatin 75 mg/m^2^ on day 1;
(2) GP regimen: gemcitabine 1250 mg/m^2^ on days 1 and 8, and cisplatin 75 mg/m^2^ on day 1;
(3) TC regimen: paclitaxel 200 mg/m^2^ on day 1, and carboplatin area under the curve 6 on day 1;
(4) DP regimen: docetaxel 75 mg/m^2^ on day 1, and cisplatin 75 mg/m^2^ on day 1.

Any questions raised by the participants will be answered to promote completion. The detailed study schedule is listed in Table 2.

**Table 2:**
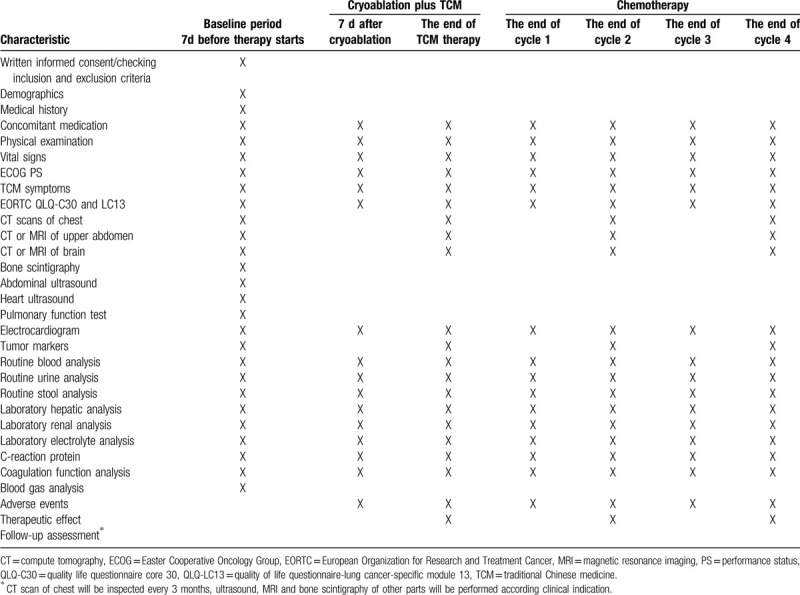
Data collection schedule.

### 2.9 Outcome measurements

Outcome measurements are scheduled at screening, baseline, and every visit.

#### 2.9.1 Basic characteristic variables

Basic data of all the participants, including name, gender, date of birth, marital status, educational level, date of diagnosis, and medical history, will be collected. General physical examination results, including vital signs (body temperature, blood pressure, respiratory rate, and heart rate), will also be recorded. Concomitant medication will be inquired about and documented at every visit. In the follow-up period, enhanced computed tomography (CT) of chest and tumor marks will be inspected every 3 months until the deadline of the trial or death. Magnetic resonance imaging, ultrasound, bone scans, and CT scans of other parts will be inspected to assess metastasis by researchers.

#### 2.9.2 Primary outcome

The OS: Time from randomization to death for any reason. If the participants will be lost to follow-up or alive at the end of the study, the last follow-up time will be used as the death time.

#### 2.9.3 Secondary outcomes

(1) Progression-free survival: Time from the randomization to tumor progression or death. PFS rates of 6 months and 1 year will also be calculated.
(2) Objective remission rate (ORR): Enhanced CT of the chest will be performed at the baseline period and the end of therapy. The therapeutic effect will be evaluated as complete response (CR), partial response (PR), stability disease (SD), and progressive disease (PD) according to Response Evaluation Criteria in Solid Tumors (RECIST). ORR will be defined as the percentage of CR and PR cases in patients.
(3) QOL: QOL will refer to the European Organization for Research and Treatment of Cancer Quality Life Questionnaire Core 30 scale and lung cancer-specific LC13 scale.

### 2.10 Safety assessments

Safety outcomes involve blood, urine and stool analysis, hepatic and renal function, electrolyte levels, coagulation function, C-reaction protein, and electrocardiogram. Safety outcomes will be assessed at each cycle visit.

### 2.11 Quality control

Before the conduction of the protocol, a range of training will be provided to all the researchers, ensuring a full understanding and mastery of the standard operating procedures. Any change to the protocol should be reported in writing to the primary researcher in Dongfang Hospital and can be applied only after permission. The quality of CRFs will be regularly supervised by independent investigators in Dongfang Hospital. Researchers will be notified in a timely fashion if any error exists.

### 2.12 Sample size calculation

Two-sided, log-rank test of Kaplan-Meier survival analysis is used to calculate the sample size. The primary outcome is the OS. The sample size is calculated based on the primary outcome. According to the data of NCCN guidelines and previous study, the median OS is 10 months in the control group and 14 months in the study group. With an estimated 18 months of enrollment and 30 months of follow-up, we have calculated that 402 patients will be needed to show a 2-sided significance level of 5% and a statistical power of 80%. Allowing for a 10% dropout rate, the sample size has been set at 450 patients (225 in each group).

### 2.13 Data management and analysis

CRFs will be entered by 2 independent researchers and supervised by a third 1 to ensure accuracy.

Data analysis will be performed by statisticians who are blind to the randomization by using SAS9.3 software (Software security authorization No. 11202165). A significance level of 0.05 will be adopted throughout the trial. The primary outcome of the study is OS, with secondary outcomes including PFS, ORR, the incidence of adverse events, and results of a QOL survey. OS curves will be estimated for each treatment group in the full analysis set using the Kaplan-Meier method. Median OS and 95% CIs will be calculated. To evaluate differences in therapeutic effects, the HRs of the 2 groups and their 95% CIs will be calculated using the Cox proportional hazard model with adjustment factors, except for institution, included as covariates.

### 2.14 Ethics and dissemination

Central ethical approval has been confirmed from the research ethics committee of Dongfang Hospital Beijing University of Chinese Medicine (JDF-IRB-2019030103). Local ethical approval from other participating hospitals has also been obtained. The study will be conducted under the principles of the Declaration of Helsinki. The trial organization, data management, monitoring, and reporting will also be conducted under the guidelines for Good Clinical Practice and other regulations. Patients will be given full and adequate oral and written information about the study. The consent will be provided by the patient or a legally authorized representative.

## 3 Discussion

The treatment of cryoablation combined with TCM is welcomed among advanced patients in China because of the QOL. Although some studies have reported the efficiency of cryoablation combined with TCM, high-quality evidence is poor. The trial protocol is based on Standard Protocol Items: Recommendations for Interventional Trials guidelines. It has a high-quality methodology to prove whether cryoablation plus TCM has higher OS. We except that the treatment could be a better option for stage IIIB/IV NSCLC patients.

## 4 Trial status

Recruitment started in March 2019, and it is expected to finish in December 2021.

## Acknowledgments

We appreciate the efforts of all research staff participating in this trial. We also acknowledge the helpful support of all participants. The results of this clinical trial will be published in the form of academic articles by our research team.

## Author contributions

**Conceptualization**: Manqiang Sun, Kaiwen Hu.

**Data curation**: Dan Wang.

**Funding acquisition**: Kaiwen Hu.

**Methodology**: Tian Zhou.

**Project administration**: Xueni Fang.

**Resources**: Haoyue Pang.

**Supervision**: Yu Chen.
